# The Muscular Dystrophy Association’s neuroMuscular ObserVational Research Data Hub (MOVR): Design, Methods, and Initial Observations

**DOI:** 10.3233/JND-221551

**Published:** 2023-05-02

**Authors:** Elisabeth A Kilroy, Rachael Burris, Edritz Javelosa, Jessica Waits, Angela Lek, Rayne Rodgers, Hayley Opgenorth, Sharon Hesterlee

**Affiliations:** a Muscular Dystrophy Association, Chicago, IL, USA; b Kinevant Sciences, New York, NY, USA; c Axogen, Alachua, FL, USA

**Keywords:** Neuromuscular diseases, registries, muscular dystrophies, amyotrophic lateral sclerosis, spinal muscular atrophy, Duchenne muscular dystrophy, facioscapulohumeral muscular dystrophy, limb girdle muscular dystrophy, Pompe disease, Becker muscular dystrophy

## Abstract

**Background:**

Neuromuscular disease (NMD) research is experiencing tremendous growth as a result of progress in diagnostics and therapeutics yet there continues to be a significant clinical data shortage for these rare diseases. To maximize the development and impact of new therapies, the Muscular Dystrophy Association (MDA) created the neuroMuscular ObserVational Research Data Hub (MOVR) as an observational research study that collects disease-specific measures from individuals living with NMDs in the United States.

**Objective:**

This manuscript provides a description of MOVR, participants enrolled in MOVR, and longitudinal data availability.

**Methods:**

MOVR collects longitudinal data from individuals diagnosed with ALS, BMD, DMD, FSHD, LGMD, Pompe disease, or SMA, and who are seen for care at a participating MDA Care Center. Data are entered from medical records into standardized electronic case report forms (eCRFs). These eCRFs capture participants’ demographics, diagnostic journeys, clinical visits, and discontinuation from the study.

**Results:**

From January 2019 to May 2022, MOVR collected data from 50 participating care centers and 1,957 participants. Data from 1,923 participants who participated in MDA’s pilot registry were migrated into MOVR, creating a total of 3,880 participants in MOVR. Initial analysis of aggregated data demonstrated that 91% of eCRFs were complete. Forty-three percent of participants had 3 or more encounters and 50% of all encounters were 5 months or less from the previous encounter.

**Discussion:**

As a centralized data hub for multiple NMDs, MOVR serves as a platform that can be used to inform disease understanding, guide clinical trial design, and accelerate drug development for NMDs.

## INTRODUCTION

Neuromuscular diseases (NMDs) include a diverse group of rare, largely genetic conditions characterized by dysfunction of the nerves and skeletal muscles resulting in progressive muscle weakness [1, 2]. Developing meaningful treatments for NMDs is challenging, but investments in basic science research by private and public funding agencies, including over $1 billion by the Muscular Dystrophy Association (MDA) [3], have fostered improvements in drug development. Fourteen new therapies for NMDs have been approved in the last ten years and an ever-expanding pipeline of over 180 therapies are in development [3, 4].

This progress increases the need for clinicians, drug developers, regulators and payers to make data-driven efficacy and safety decisions [5]. Government regulators may require drug developers to follow recipients of novel gene transfer therapies (whether experimental or commercially approved) for at least 15 years following treatment [6]. Similarly, for expensive one-time treatments, amortized reimbursement plans may require ongoing evidence of efficacy. Further, therapies for rare diseases are more likely than non-rare diseases to receive a conditional approval, as was the case for all four exon-skipping therapies for Duchenne muscular dystrophy (DMD). These conditional approvals require follow-on studies that may be augmented by real world evidence. In the EU, from 2000 to 2019, registries served as a critical component of the approval process for 88.9% of conditionally approved orphan drugs and 100% of those approved under “exceptional circumstance” [7]. Thus, the need is greater than ever to collect and analyze clinical data for NMDs before, during, and after treatment to ensure that these drugs are meeting regulatory expectations and remain available to patients in the coming years [5].

Appreciating the value of patient data for achieving these goals, NMD-focused patient advocacy organizations and academic institutions are launching disease-specific registries with the goal of providing data to researchers and drug developers. Most of these registries to date contain patient-entered data focused on a single NMD with various levels of curation on the back end. The existing registries in this space that do contain clinic-entered data are often drug-focused registries maintained by life science companies with the goal of meeting the regulatory and payer requirements for their proprietary treatments. For example, Genzyme, a Sanofi Company and pioneer in rare disease drug commercialization, has close to 16,000 patients enrolled in its proprietary registries across four rare disease indications based on a report in 2020 [7, 8]. Unless data-sharing is enabled, other companies pursuing treatments for these same diseases would have to duplicate these efforts to access patient information and meet the same regulatory requirements.

To address this need for providing rigorous clinic-entered data in a centralized, non-biased context that is accessible to all stakeholders, MDA first launched the United States Neuromuscular Disease Registry (USNDR) in 2013 as a pilot registry to help fill the gaps in NMD data quality, quantity and availability [9]. The USNDR leveraged the nationwide multidisciplinary MDA Care Centers, a 150-site network that sees an average of 60,000 individuals annually, to collect these data. The Care Center infrastructure, combined with the strong existing relationships with principal investigators and staff, provided a clear opportunity to capture key clinical data on a reliable longitudinal basis. The USNDR collected demographic, diagnostic and clinical visit data at 26 MDA Care Centers until 2018 [9]. These data were collected from participants diagnosed with one of four disease indications: amyotrophic lateral sclerosis (ALS), Becker muscular dystrophy (BMD), DMD, and spinal muscular atrophy (SMA). The USNDR represented the first centralized registry to house data on multiple NMDs with a standardized framework for data collection and data quality assurance.

The demonstrated success and value of the pilot USNDR in the NMD field fueled the launch of the neuroMuscular ObserVational Research Data Hub (MOVR) in 2019 with an updated data collection platform that allows for easier data entry and in-line data validations [10]. MOVR now includes seven indications and 50 Care Centers. The purpose of this manuscript is to describe the design and current capabilities of MOVR focusing specifically on data collection and transformation methods as well as capabilities of data visualization and reporting. An initial analysis of the participant population is provided, as well as a discussion on how MOVR is working to continue fulfilling the unmet needs described above.

## MATERIALS AND METHODS

**Fig. 1:**
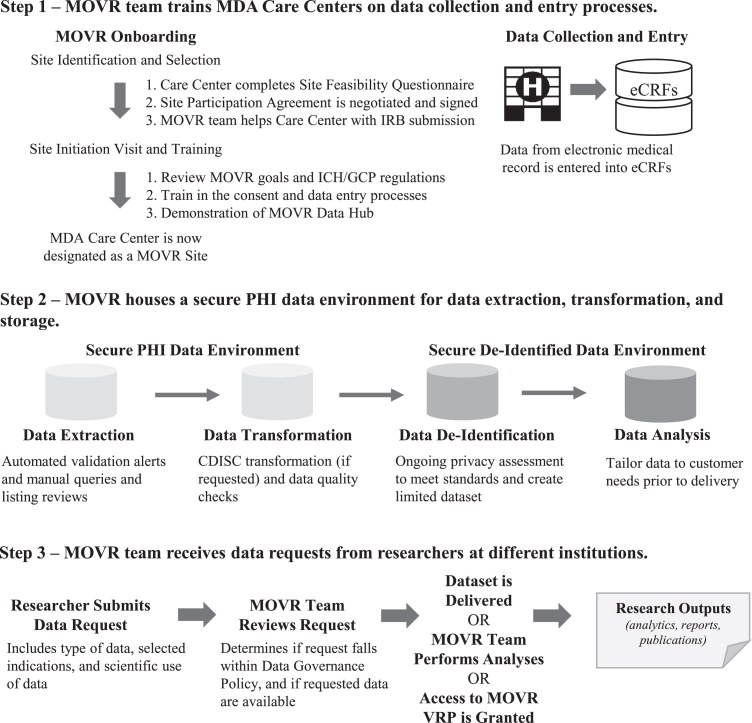
The MOVR Data Hub uses a multi-system approach for data collection, processing, and analysis. (Step 1) MDA Care Centers complete a Site Feasibility Questionnaire. Once approved by the MDA MOVR team, a Site Participation Agreement is negotiated and signed. The MDA MOVR team then provides ongoing support to MOVR Sites, including assisting with the IRB submission process, training sites how to enter data into the eCRFs, and providing answers to questions and concerns. Once training is complete, the MOVR Site begins enrolling and entering data for participants. (Step 2) Data entered in the eCRFs are extracted and transformed into a specified data format (raw vs CDISC). Data is then de-identified and stored as a limited dataset. IQVIA provides a secure PHI data environment and is responsible for data processing and quality control, standardization, and exporting. (Step 3) Researchers submit requests to access MOVR Data. Once approved, the limited dataset is customized to include only the requested data (i.e., only the indication(s) requested). The dataset is then delivered to the researcher and an active collaboration is established between the researcher and MDA MOVR team. For participating MOVR Sites, a custom visualization and reporting platform created in partnership with DNAnexus is provided for principal investigator and study staff to access and analyze data acquired by the site. Researchers may also request access to the VRP to view the aggregate dataset.

An overview of the site onboarding process, data collection and transformation processes, and accessing MOVR Data is provided in Fig. 1. A detailed discussion of each step is given below.

### Study population

Seven indications under the MDA disease umbrella were selected to participate in the initial launch of MOVR – ALS, BMD, DMD, SMA, limb girdle muscular dystrophies (LGMDs), facioscapulohumeral muscular dystrophy (FSHD), and Pompe disease. These indications were selected by considering the availability of (1) approved therapies or multiple experimental therapies in development, (2) recommended standards of care and/or extensive input from researchers and clinicians within the MDA network, and (3) existing identification and standardization of data important for clinical trials. Individuals clinically diagnosed with one of the included indications who receive care at a participating Care Center (designated as a MOVR Site) are eligible to participate in MOVR. There are no exclusion criteria nor is a confirmed genetic diagnosis required. Individuals and/or their legal guardians sign an IRB-approved written informed consent and/or assent as appropriate. It should be noted that MOVR Sites are not required to enroll participants in all seven indications. Data from the USNDR were also migrated into MOVR and USNDR participants were invited to continue contributing data in MOVR under a new consent.

### MOVR site selection & training

MOVR Sites represent a subset of MDA Care Centers who have elected to activate the MOVR Study Protocol. MDA Care Centers are specialized, multidisciplinary neuromuscular clinics funded by MDA to conduct patient visits on a regular basis (i.e., weekly, monthly).

The MDA MOVR team works collaboratively with the director of interested centers to evaluate the feasibility of participating in the MOVR Study Protocol. The MDA MOVR team then works with the center to negotiate a site contract and to receive IRB approval (from either their institutional or a central IRB) for the MOVR Study Protocol. Site onboarding by the MDA MOVR team includes training on the goals of MOVR, the International Conference on Harmonization and Good Clinical Practice regulations for observational studies, and the consent, data collection and data entry processes. Training calls are provided to new clinical research staff as needed while quarterly calls are held with each site to address questions and concerns about data entry and other MOVR-related activities. A MOVR-specific email address is monitored daily by the MDA MOVR team as a communication platform between quarterly calls.

### Data Elements and Structure

There are four electronic case report forms (eCRFs) used to input data into MOVR: diagnosis, demographics, encounter, and discontinuation. The demographics and discontinuation forms are the same for each disease indication while the diagnosis and encounter forms are unique to each indication in order to capture the disease-specific diagnostic journey and progression. Each eCRF was developed by MDA’s scientific team and a registry-focused working group made up of experts in each disease area to ensure they aligned with site capabilities and current standards of care, while also reflecting research priorities. Continued review and improvement of these eCRFs is led by the current MOVR Research Advisory Committee (RAC).

The core data elements that are captured across all seven disease indications are listed in Table 1. The demographics eCRF captures disease type, enrollment date, gender, DOB, race and ethnicity, insurance, education, and employment. The diagnosis eCRF captures the date and age at diagnosis, symptom onset, genetic testing results, family history, first symptoms of disease, and clinical sub-diagnosis (i.e., LGMD2i, FSHD1, SMA Type I, etc.). The encounter eCRF captures longitudinal data on disease progression, including the encounter date, height and weight, clinical trial participation, surgical history, hospitalizations, functional measures, pulmonary and assistive devices, and multidisciplinary care referrals. Finally, the discontinuation eCRF captures the cause and date for discontinuation, and if the cause was due to the participant becoming deceased, additional details including cause and date of death are captured. In addition to these core data elements, there are additional data elements that are specific to certain indications (Table 2). For example, muscle biopsy information is captured for DMD, BMD, and LGMD but not for the other indications, while method of diagnosis (i.e., newborn screening, clinical presentation of symptoms, etc.) are captured for Pompe disease and SMA but not for the other indications. Each eCRF contains specific data fields that are marked as required and must be filled out for the form to be completed.

**Table 1:** Core data elements captured by the electronic case report forms

Demographics eCRF	Diagnosis eCRF^*^	Encounter eCRF^*^	Discontinuation eCRF
*(During Enrollment)*	*(During Enrollment)*	*(During Enrollment and Clinical Visits)*	*(After End of Study)*
Disease Type	Age at Diagnosis	Encounter Date	Date of Discontinuation
Enrollment Date	Age at Symptom Onset	Height	Reason for Discontinuation
Gender	Clinical Diagnosis	Weight	Date of Death
DOB	First Symptoms	Clinical Trial Participation	Cause of Death
Race	Family History	Surgeries
Ethnicity	Genetic Testing Results	Hospitalizations
Insurance		Medications
Education		Pulmonary Devices
Employment		Assistive Devices
		Functional Mobility Tests
		Pulmonary Tests
		Referral Types

^*^Diagnosis and Encounter eCRFs contain additional unique fields for each indication.

**Table 2:** Data elements captured by electronic case report forms that are unique to each indication

ALS	BMD and DMD	FSHD
Revised El Escorial Criteria	Muscle Biopsy	4q35 Deletions
Body Regions First Affected	Gross Motor Milestones	4qA/4qB Haplotype Test
Number of Falls	Glucocorticoid Use	D4Z4 Methylation Assay
ALSFRS-R	Glucocorticoid Complications	SMCHD1 Sequencing
Mental Status	Scoliosis	FSHD Clinical Score
Nutritional Therapies	Cardiology Tests	Vignos Scale Grades
End of Life Planning	Nutritional Therapies	Spinal Conditions

LGMD	Pompe	SMA
Muscle Biopsy	Diagnosis Method	Diagnosis Method
Number of Falls	Pre-Symptomatic or Symptomatic	SMN1 Copy Number
Vignos Scale Grades	GAA Enzyme Activity	SMN2 Copy Number
Scoliosis	Cross-Reactive Immunologic Material (CRIM) Status	Gross Motor Milestones
Cardiology Tests	Enzyme Replacement Therapy	CHOP-INTEND
Nutritional Therapies	Gross Motor Milestones	HFMS-E
	Vignos Scale Grades	Scoliosis
	Scoliosis	Nutritional Therapies
	ENT and Hearing	End of Life Planning
	Cardiology Tests	Spinraza Treatment Log
	Nutritional Therapies	Ventilation Treatments
	Neuroimaging

### Data collection

The eCRFs are housed on a web-based portal hosted by IQVIA’s Registry Platform (IRP). Before beginning the study, the PI at a MOVR Site completes a Delegation of Authority log, which documents which individuals are responsible for data entry. Typically, data entry is completed by a research coordinator and/or a research nurse. Clinic study staff at a MOVR Site complete the eCRFs using data available from the participant’s electronic health record (EHR). MOVR Sites also have the capability of using EHR integration and/or batch data entry to auto-populate the eCRFs, but at time of publication, no sites have elected to use this method. The diagnosis, demographics and encounter eCRFs are completed at the initial study enrollment visit. Additional encounter eCRFs are completed at each subsequent visit. The discontinuation eCRF is only completed if the participant withdraws from the study, is lost to follow-up for two consecutive years, or becomes deceased.

### Data quality control and processing

MOVR leverages IRP’s data quality control systems to ensure data completeness and accuracy across all MOVR Sites. Specifically, the eCRFs harbor several types of in-line data validations to reduce the entry of erroneous or implausible values. Three different levels of validation alerts indicate the severity of the problem and the action that is required to resolve the problem. For some data fields, previously entered values are highlighted to alert clinic study staff to potential errors. Extensive site training and quarterly calls are performed to discuss data entry, the quality of data being entered, and data completeness.

### Data security and privacy

In accordance with applicable federal and state laws, MOVR (through the IRP) uses industry technology standards to implement administrative, physical, and technical safeguards to protect the accessibility, confidentiality, and integrity of protected health information (PHI) and any other confidential data. Specifically, MOVR is compliant with the Health Insurance Portability and Accountability Act (HIPAA) of 1996 and its implementation requirements for PHI as well as the FDA 21 Code of Federal Regulations Part 11 standards, which governs the proper use of electronic records and electronic signatures. The IRP undergoes annual independent HIPAA risk assessments [11] and an International Organization for Standardization 27001 certification process [12].

Participant privacy is ensured by user access controls that are actively monitored on a quarterly basis. Only authorized personnel at a MOVR Site have access to the fully identified data collected from participants at their site. Personnel from one site are not able to view identifiable data from participants enrolled at another site. A limited aggregate dataset for MDA and its third party service providers is de-identified following the guidelines defined in the Privacy Regulations issued under the HIPAA Privacy Rule [13]. For external data requesters, including academic and non-academic researchers and clinicians, a de-identification solution was identified in partnership with Privacy Analytics, an IQVIA Company, through the use of a HIPAA Expert Determination analysis [14], ensuring the highest utility of data while protecting the privacy of participants.

### Data availability and governance

The overarching purpose of MOVR is to provide clinic-entered data to researchers and clinicians. A Data Governance Policy defines (1) MDA’s roles and responsibilities, (2) authorized and non-authorized use of data, (3) prohibited use of data, (4) data ownership, (5) publication guidelines, and (5) applicable fees. The Data Governance Policy must be read and agreed to before requesting access to MOVR Data and the Data Access, Use and Distribution Agreement must be signed prior to MOVR Data being delivered.

MOVR Data may be requested by any person located in the United States or abroad for an authorized use as described in the Data Governance Policy, including representatives from MOVR Sites, academia, industry, government, or nonprofit organizations. Data requests follow a formal review process to ensure that requested data is available within MOVR and that the proposed project design and analyses will produce meaningful scientific findings. Researchers must ensure that MOVR Data is stored securely using software that is compliant with current laws and regulations and is kept up-to-date. MOVR Sites and other selected researchers can also conduct analyses and visualize MOVR Data in a custom Visualization and Reporting Platform (VRP) hosted by DNAnexus. A description of the MOVR VRP is provided below (see “MOVR Visualization and Reporting Platform”). Finally, MOVR Data can be transformed into the Clinical Data Interchange Standards Consortium (CDISC) standard format [15] if requested. The MOVR RAC reviews any requests that fall outside of authorized and non-authorized uses and is responsible for making the final decision as to whether access to data will be granted.

### MOVR visualization and reporting platform

The MOVR VRP enables MOVR Sites to visualize, organize, analyze, and report on their site-specific data and, if requested using the steps described above, the de-identified aggregate data. Approved researchers may also access MOVR Data via the VRP. Researchers can easily filter the MOVR dataset by any data field and save these filtered datasets as cohorts, which can be shared within research groups. Researchers are able to perform additional analyses by creating JupyterLab environments backed by Spark clusters to directly query MOVR Data and create data frames within a Python or R environment. Custom workflows and apps can also be created to perform routine analytics as MOVR Data is updated. To ensure the successful use of the MOVR VRP, new users can enroll in virtual live demos and have access to the MDA Community Project Portal, which includes newsletters, training guides and videos, FAQs, and a direct support link to DNAnexus, who hosts and manages the VRP.

### Initial data analysis

MOVR Data were aggregated in May 2022 for preparation of this manuscript. Custom scripts using Python 3.10.0 and the NumPy 1.21.4 [16] and Pandas 1.3.4 [17, 18] libraries were written to generate the datasets used to perform the below analyses. The Matplotlib [19] and Seaborn [20] libraries were used to generate the graphical representations derived from these datasets.

Participant enrollment was determined by counting those participants who had a demographics, diagnosis, and at least one encounter eCRF. An index was created for these participants and used in all analyses so that only those participants who met this criterion were included. Two sub-indices were created to distinguish those participants who are enrolled in MOVR versus those whose data were migrated from USNDR (also referred to as ‘Legacy’).

The genetic confirmation data field is completed by selecting one of five categories with the exception of ALS, which has an additional ‘Other’ category. For this analysis, the number of participants within each category was counted and totaled for each indication.

For the sex assigned at birth and the ethnicity analyses, the number of participants in each category for the respective data field was counted and totaled for each indication. For the race and insurance type data fields, multiple categories can be selected. For participants with multiple categories selected, a new category (either ‘Multi-Racial’ or ‘Mixed Sources’ category, respectively) was assigned and then each category was counted and totaled for each indication. For all analyses, missing data fields and data fields containing ‘Not Reported’ or ‘Unknown’ were combined into a single category entitled ‘Unknown’.

The age at diagnosis data field was used to calculate the mean, median, the lower quartile (Q1), and the upper quartile (Q3). The interquartile range (IQR) was then calculated and used to identify outliers. Boxplots were created to visualize these data for each indication.

Longitudinal data availability was determined using three different measures: (1) the total number of encounters for each participant, (2) the time between a participant’s first encounter and their most recent encounter, and (3) the time between consecutive encounters. For the first measure, the number of encounters per participant was calculated and then assigned to one of five bins. The total number of participants assigned to a bin was then counted and totaled for each indication. For the second measure, the number of months between a participant’s first and most recent encounter was calculated and then assigned to one of six bins. The number of participants assigned to a bin was then counted and totaled for each indication. Finally, for the third measure, the number of months between each consecutive encounter for a participant was calculated and then assigned to one of six bins. The number of encounters within each bin was then counted and totaled for each indication. The average number of months between the first and most recent encounter as well as the average number of months between consecutive encounters were also calculated for each indication. Note, for the second and third measure of longitudinal data availability, participants who only had one encounter were not included in these two analyses.

Form completeness was introduced when MOVR was launched and did not exist in the USNDR dataset. Therefore, only those forms submitted for participants enrolled in the MOVR Study Protocol were used to assess form completeness. The number of eCRFs marked as ‘Complete’ or as ‘Incomplete’ were counted and totaled for each of the four eCRFs across the seven indications.

## RESULTS

### MOVR sites have actively enrolled participants across 7 NMDs

**Fig. 2:**
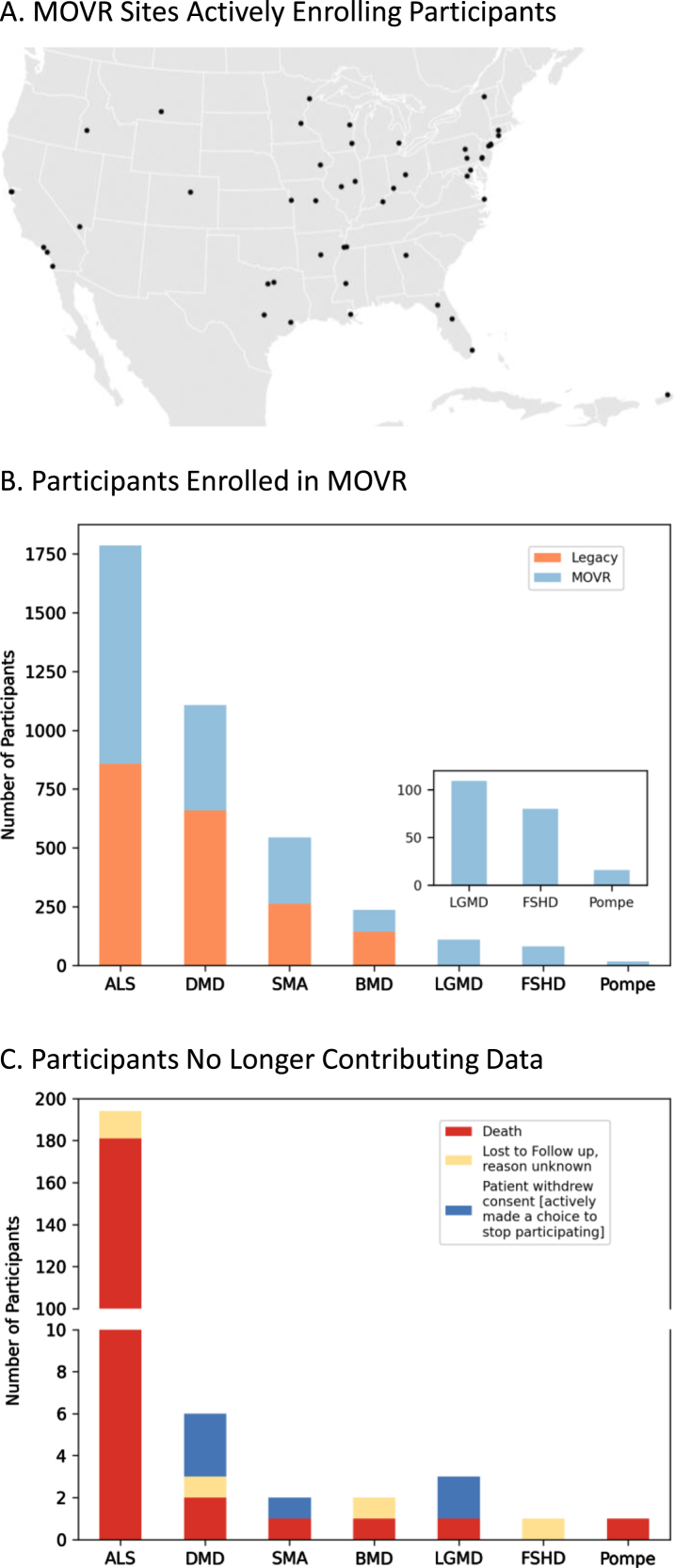
MOVR sites have actively enrolled participants across 7 NMDs. (A) Participants enrolled in MOVR vs those whose data were migrated from the legacy USNDR study. The total number of participants for ALS, DMD, SMA, BMD, LGMD, FSHD, and Pompe is 1,787, 1,107, 544, 237, 109, 80, and 16 respectively. Note that LGMD, FSHD, and Pompe were not collected in the USNDR dataset. (B) Participants who were no longer contributing data due to death, lost to follow up, or withdrawing consent. The number of participants no longer contributing data into MOVR was 209 (*n* = 194 ALS, *n* = 6 DMD, *n* = 2 SMA, *n* = 2 BMD, *n* = 3 LGMD, *n* = 1 FSHD, and *n* = 1 Pompe).

Most rare disease registries are powered by patients and their families who provide patient reported outcomes (PROs). Conversely, MOVR leverages the MDA Care Center network which is comprised of over 150 care centers and 300 NMDs across the United States to gather clinic-entered data. MOVR has onboarded 50 sites across 31 states (Fig. 2A). These sites were classified as adult only (*n* = 9), pediatric only (*n* = 16), ALS only (*n* = 4), adult and pediatric (*n* = 11), and adult and ALS (*n* = 10) Care Centers. Thirty-four MOVR Sites use the central IRB while 16 sites use their local/institutional IRB. The combined USNDR and MOVR data hub has 3,880 participants living with: ALS (*n* = 1,787), DMD (*n* = 1,107), SMA (*n* = 544), BMD (*n* = 237), LGMD (*n* = 109), FSHD (*n* = 80), and Pompe (*n* = 16) (Fig. 2B). Of these participants, 1,957 were enrolled directly into MOVR since 2019, while data from 1,923 participants were migrated from the USNDR (Fig. 2B). A total of 209 participants are no longer actively participating in MOVR (Fig. 2C) due to a combination of death (*n* = 187; 89%), withdrawal of consent (*n* = 6; 3%) or lost to follow-up (*n* = 16; 8%). The majority of participants who are no longer contributing data were those with ALS (*n* = 181; 87%) who became deceased (Fig. 2B). Taken together, the MOVR data hub has the capacity to collect and analyze data from numerous care centers and thousands of enrolled participants.

### Rigorous eCRF requirements have resulted in a greater than 90% completion rate for MOVR only data

**Fig. 3:**
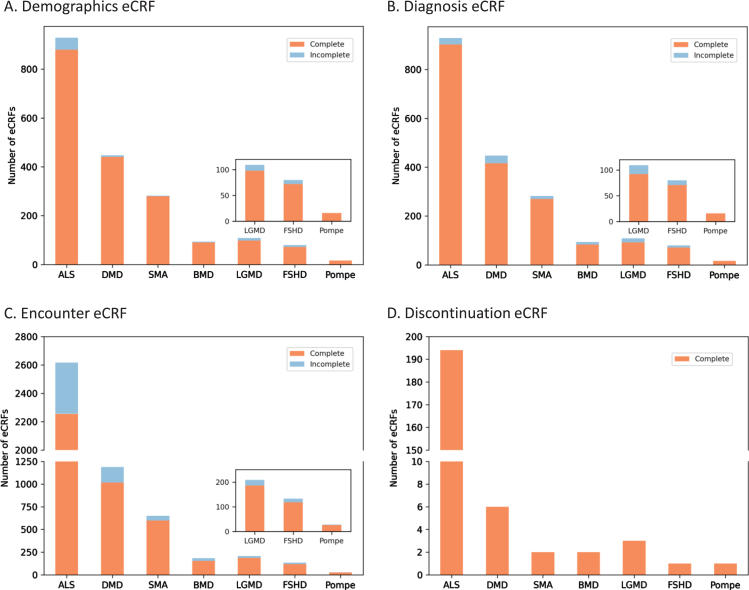
MOVR Form completeness. Form completeness for (A) Demographic eCRFs, (B) Diagnosis CRFs, and (C) Encounter eCRFs were 96, 95, and 87 percent complete, respectively. (D) One hundred percent of Discontinuation eCRFs were completed for all seven indications.

USNDR data entry protocols did not include in-line validations nor form completion guidelines. To improve data quality, MOVR implemented over 250 required fields across the eCRFs that must be completed, meaning over 75% of the main questions (excludes dependencies and sub-questions) must be answered. For MOVR only data demographic eCRFs were found to be 96% completed (Fig. 3A), diagnosis eCRF were 95% completed (Fig. 3B), and encounter eCRF were 87% completed, with the majority of the incomplete forms for ALS participants (Fig. 3C). Finally, 100% of the discontinuation eCRFs were completed (Fig. 3D). Combined, this means that 91% of all MOVR eCRFs were marked complete. The implementation of required fields has created a core dataset for each participant in MOVR, allowing a complete understanding of participant demographics, diagnostic journey, and disease progression across all MOVR sites.

### The majority of MOVR participants have a confirmed laboratory genetic diagnosis

**Fig. 4:**
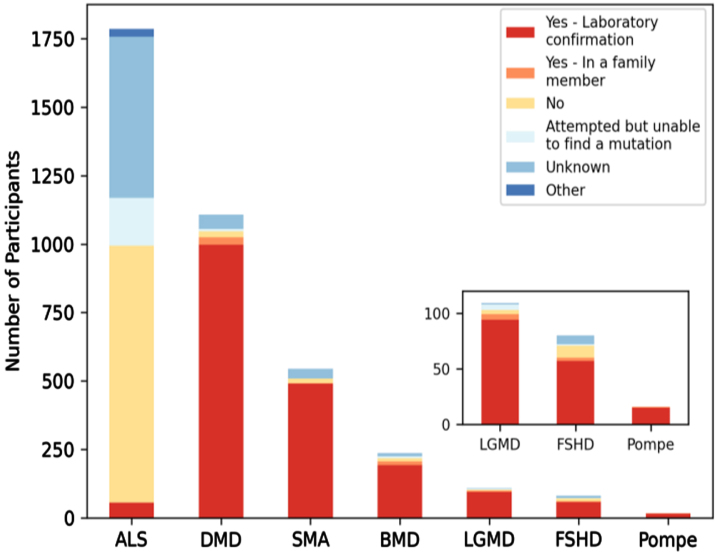
The majority of MOVR participants have a confirmed laboratory genetic diagnosis. Six of the 7 indications in MOVR are caused by a genetic mutation with the exception of ALS. Ninety percent of participants with DMD or SMA had a laboratory confirmed genetic diagnosis while 81% of participants with BMD, 71% with FSHD, 86% with LGMD, and 94% of those with Pompe disease have a laboratory confirmed genetic diagnosis. These percentages increase when including those participants who have an affected family member. For ALS, only 3% of participants of a confirmed laboratory diagnosis.

Neuromuscular diseases are difficult to genetically diagnose. Participation in MOVR does not require a genetically confirmed diagnosis nor does the MOVR Study Protocol implement a standardized set of diagnostic criteria that must be met. Rather, a participant’s diagnosis is dependent upon the expertise of the clinicians at the participating care centers. Excluding participants with ALS, 88% of MOVR participants have a confirmed laboratory diagnosis (Fig. 4).

### MOVR is comprised of participants of varying sexes and insurance backgrounds but does not fully reflect the racial and ethnic diversity of the United States Population

**Fig. 5:**
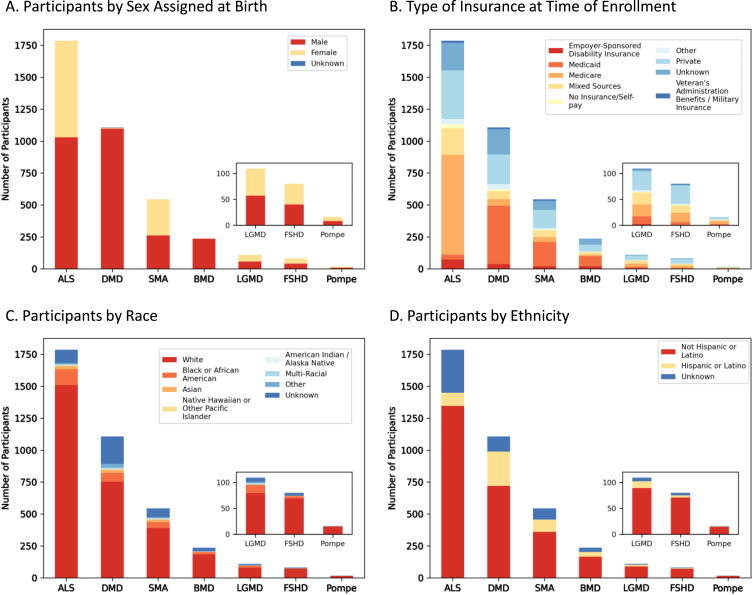
Demographic composition of MOVR participants. (A) Number of participants assigned male or female at birth. (B) Number of participants with different types of insurance at time of enrollment. Note, ‘Mixed Sources’ represents those participants who have more than one type of insurance. (C) Number of participants by race, where ‘Multi-Racial’ represents those participants with more than one race selected. (D) Number of participants by ethnicity.

Demographic analyses performed on MOVR participants identified a nearly equal distribution of persons assigned male or female at birth with ALS (*n* = 1,030 M, *n* = 757 F), SMA (*n* = 262 M, *n* = 282 F), LGMD (*n* = 57 M, *n* = 52 F), FSHD (*n* = 40 M, *n* = 40 F), and Pompe (*n* = 8 M, *n* = 8 F) (Fig. 5A). Most participants with DMD and BMD were male (*n* = 1,097 and *n* = 236, respectively) while 7 participants with DMD were female and 3 were unknown (Fig. 5A). For BMD, only 1 participant was marked as unknown (Fig. 5A). The type of insurance for participants is variable across the seven indications. For DMD, SMA, and BMD, Medicaid is the most common insurance type with 41, 35, and 34% of participants, respectively, having Medicaid as their only insurance (Fig. 5B). For participants with ALS and Pompe, Medicare is the most common insurance type with 44 and 38% of participants, respectively, having Medicare as their only insurance (Fig. 5B). Medicare and Medicaid together comprise most of the insurances with 44% of all MOVR participants having either Medicare or Medicaid. Only 23% of MOVR participants have private insurance while 1% of participants have no insurance/self-pay (Fig. 5B). Both race (Fig. 5C) and ethnicity (Fig. 5D) were analyzed for each indication. The majority of participants identify as White (77% Fig. 5C). Race was unknown for 11% of participants (Fig. 5C). Ethnically, participants identify as Non-Hispanic or Latino (71%) or Hispanic or Latino (13%; Fig. 5D). Ethnicity was unknown for 15% of participants (Fig. 5D).

### The age at diagnosis of MOVR participants is variable within each of the 7 indications

**Fig. 6:**
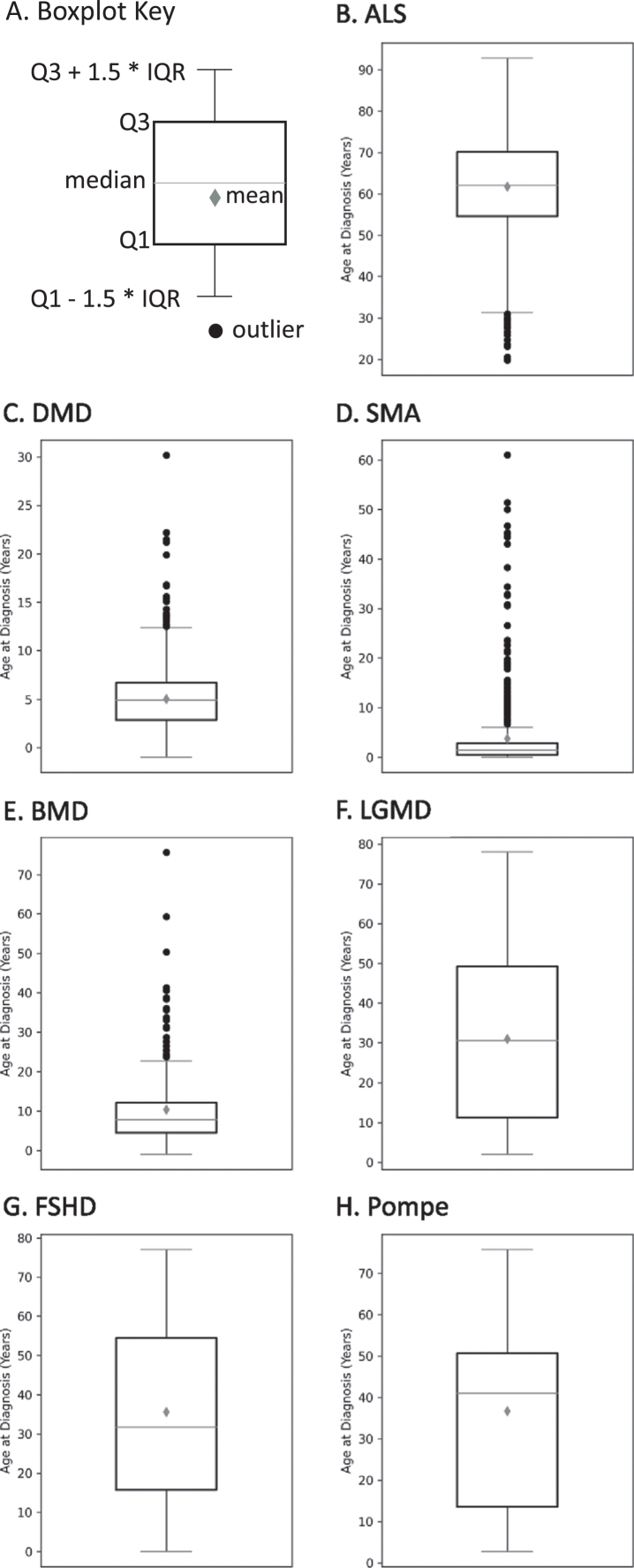
The age at diagnosis of MOVR participants. (A) Boxplot key highlights key parameters used to build boxplots. Q1 and Q3 represent 25 and 75 percent, respectively. IQR is the interquartile range. The gray diamond is the mean while the horizontal line across the box is the median. Black circles represent outliers. Boxplots are shown for (B) ALS, (C) DMD, (D) SMA, (E) BMD, (F) LGMD, (G) FSHD, and (H) Pompe.

As shown in Fig. 6, age at diagnosis is variable across participants for each indication with several indications having multiple outliers. The mean age±standard deviation at diagnosis was 61.8±11.6 years for ALS (Fig. 6B), 5.0±3.1 years for DMD (Fig. 6C), 3.8±8.0 years for SMA (Fig. 6D), 10.3±10.2 years for BMD (Fig. 6E), 30.9±21.0 years for LGMD (Fig. 6F), 35.6±21.6 years for FSHD (Fig. 6G), and 36.7±25.4 years for Pompe (Fig. 6H). For those indications with multiple subclinical diagnoses/classifications, variability for age at diagnosis was reduced (data not shown).

### MOVR has the capacity for longitudinal data collection across disease progression through clinical encounters

**Fig. 7:**
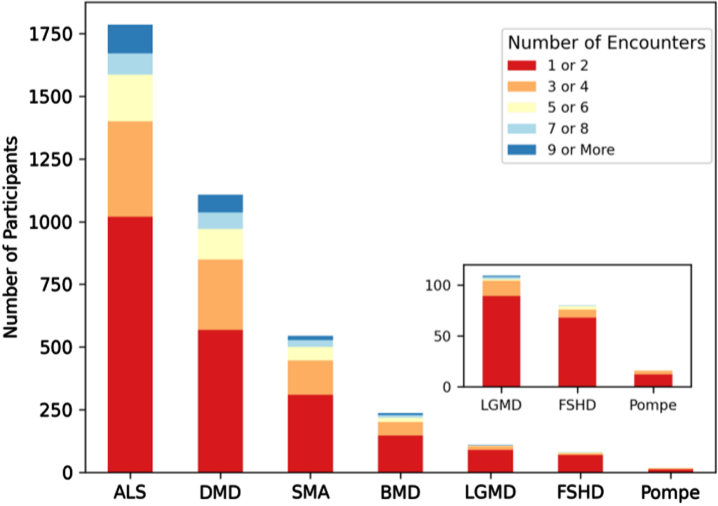
MOVR has the capacity for longitudinal data collection across disease progression through clinical encounters. Longitudinal data availability in MOVR was calculated by the number of encounters per participant and then assigned to one of five groups.

Longitudinal data availability was assessed using three different metrics. The first metric assessed was the number of encounters per MOVR participant (Fig. 7). Fifty-seven percent of participants had 1 to 2 encounters captured in MOVR while 23 percent had 3 to 4 encounters. Twenty percent of participants had 5 or more encounters. The mean number of encounters per participant was 3.2 for ALS, 3.3 for DMD, 2.9 for SMA, 2.7 for BMD, 1.9 for LGMD, 1.7 for FSHD, and 1.8 for Pompe. The average number of months between the first and most recent encounter ranges from 13.7 months to 26.5 months. Some participants (27%) had data collected across 26 or more months (Table 3). The average number of months between consecutive encounters ranges from 3.9 months to 9.1 months (Table 3), and 50% of these encounters took place within 5 or less months of each other. Taken together, these data provide MOVR with the capacity for capturing disease progression at routine clinical visits at multi-disciplinary NMD clinics.

**Table 3:** Longitudinal data availability across the seven indications

*Time Between First and Most Recent Encounter^1^*							
	Average (Months)	5 or Less Months^2^	6 to 10 Months^2^	11 to 25 Months^2^	16 to 20 Months^2^	21 to 25 Months^2^	26 or More Months^2^
ALS	13.7	315	271	167	117	74	153
DMD	26.5	20	170	99	71	87	309
SMA	23.4	25	87	42	33	25	128
BMD	26.0	8	23	24	20	9	54
LGMD	14.8	2	14	21	6	3	1
FSHD	14.3	2	6	9	4	7	1
Pompe	14.3	0	1	3	2	1	0
*Time Between Consecutive Encounters^1^*							
	Average (Months)	5 or Less Months^3^	6 to 10 Months^3^	11 to 25 Months^3^	16 to 20 Months^3^	21 to 25 Months^3^	26 or More Months^3^
ALS	3.9	3163	594	49	23	11	40
DMD	7.9	435	1677	271	71	44	50
SMA	7.8	312	512	113	41	15	28
BMD	9.1	44	235	88	10	4	13
LGMD	7.2	34	40	21	2	2	0
FSHD	7.8	13	24	13	1	1	1
Pompe	8.4	2	4	6	0	0	0

^1^Only includes participants with at least 2 encounters. ^2^Represents number of participants. ^3^Represents number of encounters.

## DISCUSSION

MOVR was created to address the significant data shortage for NMDs. It is the first data hub to collect longitudinal, clinic-entered data from participants living with one of seven NMDs. The wide range of geographical locations of MOVR sites paired with the largely completed eCRFs combine to make MOVR a powerful data hub for studying disease progression, understanding the relationships between phenotype and genotype, and for conducting clinical trial feasibility analyses. A comprehensive analysis of every participant is critical for the study of rare diseases, as each participant can offer valuable insight into disease progression and clinical interventions. Additionally, as registries become more prominent, individual patient data can be used to design individual treatment plans and make real-time decisions. Specifically, MOVR is providing clinicians with access to their patient data and the opportunity to view their patient’s individual disease progression against data captured across all MOVR Sites from individuals diagnosed with the same disease. Education centered on leveraging large datasets is becoming an important component of the neuromuscular disease field.

While a laboratory confirmed genetic diagnosis is not required for participation in MOVR, the majority of MOVR participants with the exception of those with ALS have a laboratory confirmation. This includes participants living with LGMD, where 86% of participants have an identified gene causing their muscle wasting and weakness. This value is drastically different from previously published studies, where only 52% [21] and 27% [22] of individuals presenting with an LGMD-like phenotype had an established molecular diagnosis. These results suggest that there may be an ascertainment bias across MOVR sites such that sites are more likely to enroll participants who have a confirmed genetic diagnosis even though it is not required for participation.

Sex distribution and age at diagnosis across MOVR participants are similar to those published by other registries and clinical reports for where data are available, and only small differences arise due to how metrics are defined [23–29]. Sex assigned at birth is consistent with previously published data for FSHD, Pompe disease and SMA, which have relatively equal numbers of males and females affected [24, 26, 28]. For ALS, there are more males than females in MOVR, which is consistent with the National ALS registry [25]. As DMD and BMD are X-linked recessive diseases, it is expected that primarily males are present in MOVR and this is also the case in previously published datasets; however, there are 9 females diagnosed with DMD in MOVR. It is estimated that up to 7.8% of females harboring mutations in the *DMD* gene are manifesting carriers, developing symptoms that may range from mild muscle weakness to a rapidly progressive DMD-like muscular dystrophy [30]. Future analyses can be conducted on these participants to better understand their disease progression. Finally, NMDs exhibit a spectrum of disease onset and severity, which can lead to large ranges in the age at diagnosis. The average ages at diagnosis for each indication ( Fig. 6) were consistent with previously published data [23–29]. For example, the average age at diagnosis for MOVR participants with ALS is 61.8±11.6 years, which is comparable to the average of 64.4±2.9 years reported in a systematic review on the global epidemiology of ALS [29] and within the range of 60 to 69 years which is reported by the National ALS registry as the age range with the highest percentage of cases [25]. This is also similar for individuals with DMD and BMD, as the average age at diagnosis is 5.0±3.1 years for DMD and 10.3±10.2 years for BMD in MOVR compared to 4.9±1.7 years [23] and 10 years [27] respectively in published registry datasets.

As discussed previously, the goal of MOVR is to efficiently capture clinical data from multidisciplinary visits happening throughout the MDA Care Center Network. To evaluate the potential of MOVR to capture NMD data across the United States, we quantified (1) the number of MOVR sites actively entering data, (2) the distribution of these sites across the United States, (3) the number of enrolled participants, and (4) the completeness of entered data.

The MDA leverages its Care Center network and investigator relationships to collect disease-centric data across seven indications. MOVR expands and improves upon the pilot USNDR registry, providing a more rigorous data management system and visualization platform that ensures data is of value to researchers, clinicians, drug developers and regulators. The value of MOVR, or any clinic-entered patient database, is dependent on the quantity, completeness, quality and representativeness of data. The results described above demonstrate how MOVR can drive therapeutic development and satisfy regulatory requirements by fulfilling these four database requirements.

### Quantity

Since its inception in 2019 through the cut-off date for this publication, the MOVR Data Hub has collected data from 1,957 participants across seven indications from 50 sites, for a total of 3,880 participants when combined with legacy USNDR data. MDA’s goal is to extend MOVR to the majority of its 150 Care Centers, which collectively see over 60,000 individuals annually for regular care and participation in trials, and to add additional indications. This growth to date and potential for scale places MOVR in a prime position to the meet the data needs of investigators and drug developers. Even though the COVID-19 pandemic has disrupted clinical visits, MOVR continued to see growth as a direct result of enhancements such as the ability to consent participants remotely.

To increase the number of participants and the longitudinal data available in MOVR, the MDA MOVR team is working with sites to develop strategies that ease the burden of manual data entry on site staff. Currently, EHR integration is an available resource offered to participating MOVR Sites, but it requires collaboration between each site’s Information Technology department and IQVIA. Feedback received from sites so far suggests that manual data entry is preferred due to limited resources and conflicting priorities, although a few newer sites are now attempting to implement EHR integration. Many of the data elements captured in MOVR are not standardized fields in the health record and are found in clinical notes, which is a major barrier to successful EHR integration.

### Completeness

The completeness of data in MOVR is generally very high with around 90% of eCRFs having all required fields completed. This being said, the MDA MOVR team continues to work closely with site personnel to enhance the completeness of data, including fields that are not listed as “required.” For example, the COVID-19 pandemic prevented some required functional tests such as pulmonary measurements from being performed during encounter visits, resulting in the inability to enter these data. The MDA MOVR team is also evaluating whether some data are systematically more likely to be missing due to inconsistencies in how data are reported in the EHR verses the wording of the question in the eCRFs.

### Quality

Data quality is defined by the MDA MOVR team as data that is accurate and serves the purpose of advancing NMD research. Although MOVR has several processes in place to ensure that data entered into MOVR reflects the medical record, a systematic source document verification audit of MOVR Data and medical records will be completed to truly evaluate the accuracy of these data. Source document verification is planned for 2022 and a more detailed exploration of data quality will be published separately.

The MDA MOVR team also assessed how compliant MOVR is according to the recently published draft FDA guidance on assessing registries to support regulatory-decision making for drug and biological products [31]. Seven key topics were discussed in the document, including data dictionary, rules for data validations, data quality assessments and auditing, procedures for data collection, curation, management and storage, data access, data protection and security, version control, and updating eCRFs to reflect changing clinical information. MOVR meets 24 out of 31 guidelines (77%) discussed in the draft document and is currently implementing and/or working on strategies to address those guidelines that were not satisfied.

### Representativeness

Patient registries often struggle achieving a representative and inclusive population, which limits the ability to accelerate research and clinical trials [32]. Datasets that do not represent a patient population as a whole are recognized as problematic in recent regulatory guidance from both the FDA and the EMA, which require that sources of bias be identified and documented if registry data are to be used as the basis of regulatory decisions [31, 33]. Our analysis of MOVR Data indicates that the MOVR participant population is not representative of the racially and ethnically diverse population of the United States, despite the indications captured by MOVR having no known ethnic biases in their incidence. Specifically, according to census data from 2021, the United States population is 59.3% White, 18.9% Hispanic, 13.6% Black and 6.1% Asian [34]. MOVR participants are 77% White, 7% Black or African American, and 2% Asian and 13% identify as Hispanic or Latino. It is not yet clear if the bias towards White, non-Hispanic participants reflects the (1) patient population at MOVR Sites, (2) the selection of patients offered participation in MOVR, and/or (3) inequalities in access to healthcare. The MDA MOVR team is developing a plan to better understand the representativeness of MOVR Data as this is a high priority for MDA and its goal of using MOVR Data to support regulatory submissions. Additionally, while data collection is currently limited to multidisciplinary care centers across the United States, expanding the MOVR Study Protocol to care centers around the world could be extremely beneficial in increasing the representativeness of the data as well as provide an opportunity to understand diagnostic journeys and disease progression at a global level. Global expansion is something that may be considered in the future after further build-out in the United States.

To add dimensionality to the data, MOVR plans to integrate patient-reported outcomes (PROs) with the existing clinic-entered dataset. PROs offer unique insights into natural history, disease progression, quality of life, and experience from the perspective of the participant [35]. When possible, PROs for individual indications will be integrated through collaborations with existing third-party registries, and where this is not possible MOVR will develop PROs de novo. MOVR is also launching a Community Advisory Committee, which will serve as a platform for MOVR participants to provide feedback on the relevance of data collected to the lived experience of NMDs, on MOVR priorities for new features and indications, and develop mechanisms that measure the success of MOVR from the participant and family perspectives. Finally, MOVR will gradually expand the number of indications to further support research in the NMD field.

### Conclusion

In a fast-paced environment where the needs of drug developers and care providers are changing quickly, access to high quality clinical and patient data is essential. MOVR is a versatile and rigorous data platform designed to ensure that data from every clinic visit at participating MDA Care Centers are captured in a format that can be used for a variety of purposes. Ultimately, the existence of such a neuromuscular disease registry that is national in scope may help forestall the development of proprietary industry databases and siloing of data in the rare disease space.

**Table:** 

MOVR Site^*^	Site PI
Arkansas Children’s Research Institute	Aravindhan Veerapandiyan
Beaumont Health, Royal Oak	Meghan Harper-Shankie
Billings Clinic	Steven Arbogast
Carle Physician Group	Robert Cranston
Child Neurology Consultants of Austin	Meeta Cardon
Children’s Hospital Colorado	Susan Apkon
Children’s Hospital Los Angeles	Leigh Ramos-Platt
Children’s Hospital of The King’s Daughters	Crystal Proud
Children’s Hospital of Wisconsin	Matthew Harmelink
Cincinnati Children’s Hospital Medical Center	Cuixia Tian
Columbia University Medical Center	Michio Hirano
Cook Children’s Medical Center	Warren Marks
Essentia Institute of Rural Health	Amber Erickson
Geisinger Medical Center	Jose Avila
Georgetown University Hospital	Shakti Nayar
Hershey Medical Center at Penn State	Sankar Bandopadhyay
Hospital for Special Care	Kevin Felice
Hospital for Special Surgery	Dale Lange
Houston Methodist Neurological Institute	Ericka Greene
Idaho Physical Medicine and Rehabilitation	Robert Friedman
Johns Hopkins University	Lora Clawson
Las Vegas Clinic, McKinnon Medical Group	Jonathan McKinnon
Le Bonheur Children’s Hospital	Elena Caron
Louisiana State University Health Science Center NOLA	Deidre Devier
Massachusetts General Hospital	James Berry
Medical College of Wisconsin (Froedtert)	Michael Collins
Medical University of South Carolina	I-Hweii Amy Chen
Nemours Children’s Hospital	Migvis Monduy
Ohio State University	Bakri Elsheikh
Rady Children’s Hospital (San Diego)	Chamindra Konersman
Rutgers New Jersey Medical School	Nizar Souayah
Southern Illinois University Medical Center	James Gilchrist
St. Vincent Hospital at Prevea Health	Terence Edgar
Temple University Hospital	Terry Heiman-Patterson
Texas Neurology	Daragh Heitzman
UCSF Benioff Children’s Hospital	Jonathan Strober
University of California, Los Angeles	Perry Shieh
University of California, San Francisco Medical Center	Ann Poncelet
University of Florida Health (Pediatrics)	Barry Byrne
University of Iowa Hospitals and Clinics	Katherine Mathews
University of Kansas Medical Center	Jeffrey Statland
University of Louisville (Pediatrics)	Arpita Lakhotia
University of Miami Kessenich Family Center	Mario Saporta
University of Minnesota	Peter Karachunski
University of Mississippi Medical Center	Amanda Witt
University of Missouri	Raghav Govindarajan
University of Puerto Rico Medical Sciences	Brenda Deliz
University of Vermont Medical Center	Waqar Waheed
Wesley Neurology Clinic, PC,	Tulio Bertorini
Yale School of Medicine – Yale New Haven Children’s	Bhaskar Roy, Cristian Ionita
Pediatric Specialty Center

^*^These are the sites that were active and contributed data used at the time of writing the manuscript.

## DATASETS AND DATA ARTICLES

MOVR Data is available upon request by emailing mdamovr@mdausa.org.
